# An examination of active inference in autistic adults using immersive virtual reality

**DOI:** 10.1038/s41598-021-99864-y

**Published:** 2021-10-13

**Authors:** Tom Arthur, David Harris, Gavin Buckingham, Mark Brosnan, Mark Wilson, Genevieve Williams, Sam Vine

**Affiliations:** 1 Department of Sport and Health Sciences, College of Life and Environmental Sciences, University of Exeter, St Luke’s Campus, Heavitree Road, Exeter, EX1 2LU Devon UK; 2 Centre for Applied Autism Research, Department of Psychology, University of Bath, Bath, BA2 7AY UK

**Keywords:** Autism spectrum disorders, Motor control, Sensorimotor processing, Oculomotor system

## Abstract

The integration of prior expectations, sensory information, and environmental volatility is proposed to be atypical in Autism Spectrum Disorder, yet few studies have tested these predictive processes in active movement tasks. To address this gap in the research, we used an immersive virtual-reality racquetball paradigm to explore how visual sampling behaviours and movement kinematics are adjusted in relation to unexpected, uncertain, and volatile changes in environmental statistics. We found that prior expectations concerning ball ‘bounciness’ affected sensorimotor control in both autistic and neurotypical participants, with all individuals using prediction-driven gaze strategies to track the virtual ball. However, autistic participants showed substantial differences in visuomotor behaviour when environmental conditions were more volatile. Specifically, uncertainty-related performance difficulties in these conditions were accompanied by atypical movement kinematics and visual sampling responses. Results support proposals that autistic people overestimate the volatility of sensory environments, and suggest that context-sensitive differences in active inference could explain a range of movement-related difficulties in autism.

## Introduction

Autism Spectrum Disorder (ASD, hereafter autism) is diagnosed according to atypicalities in social interaction, communication, and behavioural flexibility. However, one particular source of daily living difficulty for autistic people concerns impaired sensorimotor control^1,2^. Indeed, differences in sensory integration, motor coordination and skill acquisition are common in autistic people^2,3^, with particular impairments shown in interceptive skills like catching or hitting a ball^4–7^. These differences emerge at a kinematic level^5,7,8^, for which autistic people show noisy, inflexible, and uncertain movement patterns^7,9,10^. The degree of impairment in motor tasks correlates with an individual’s socio-behavioural traits^11^ and daily living competencies^1^. Research into the source of these sensorimotor difficulties could thus develop both our scientific understanding of autism, and our capacity to manage its various clinical features.

Neuro-computational research has shown that movement is controlled using probabilistic models about the world (i.e., *predictions*), which are derived from incoming sensory evidence and prior expectations^12,13^. When performing an action like hitting a tennis ball, the brain will regulate motor responses (e.g., movement kinematics) and sampling behaviours (e.g., gaze responses) according to both incoming sensory cues and prior beliefs (e.g., about gravity, ball bounciness^14^). Such dynamic sources of information are weighted according to their uncertainty, or *precision*, which is directly proportional to learning rate^15^. These precision-weighted predictions not only serve to optimise perceptual functions, they also represent a set point that an individual can act towards in their movements^13,16,17^. Any deviations away from near-optimal processing could thus result in sensorimotor impairment.

Indeed, various researchers have highlighted the role of impaired predictive processing in autism (see review^18^). Though conflicting in their precise explanations, most ‘simple’ Bayesian frameworks attest to an attenuated influence of prior expectations on autistic perception and action^19,20^. These accounts can explain heterogeneous socio-behavioural traits and neurological abnormalities displayed in autistic people (see clinically-focused review^21^). Furthermore, proposed differences in predictive processing^22^ align with a range of autism-related sensorimotor atypicalities, including: impaired movement planning^23,24^, reduced anticipatory motor adjustments^7,25^, suboptimal movement initiation kinematics^5,8^, slower error-based saccade adaptation^26^, and atypical gaze fixation behaviours^27^. However, prediction-related difficulties only emerge under some task conditions (see review^28^), with autistic people demonstrating intact visual motion prediction^29^, anticipatory lifting forces^30^, and non-social ocular tracking abilities^31^. These inconsistent findings undermine proposals that prior expectations are generically attenuated in autism.

As such, recent neurocomputational accounts instead argue that autism is characterised by atypicalities in precise, context-sensitive processing functions, which determine how predictive control is hierarchically adjusted according to environmental statistics (e.g., uncertainty, volatility^18,32,33^). In contrast to the simple frameworks discussed above, these mechanisms implicate how an individual *dynamically* models the world, through precision-related modulation of postsynaptic cortical gain^32,33^. Here, autistic daily living difficulties are not perceived to result from ‘one-level’ attenuations in the use of prior expectations; they are proposed to stem from mechanisms that contextually regulate prediction error across multi-level neural networks^18,32,33^. These hierarchical functions not only determine the precision (i.e., uncertainty) of prior beliefs, they also model how environmental probabilities are expected to fluctuate over time. Indeed, estimations about environmental (in)stability implicate how an individual samples and learns about sensory information from the world^15^, with even minor abnormalities likely to impair the formation of stable, statistically-optimal predictive models^33^. As a result, autistic people may consistently interact with the world as if it is uncertain or volatile^32^, a hypothesis supported by studies of probabilistic learning^34^, neural habituation^35^, and pupil diameter responses^32^.

When interpreted alongside active inference perspectives, these context-sensitive frameworks present novel, empirically-falsifiable predictions about sensorimotor behaviour^18^. According to active inference theory, optimal movement control rests on dynamic adjustments in the sampling and weighting of sensory information^12^, with physical actions used to fulfil predictions and/or reduce their uncertainty^13,16,17^. Here, the use of probabilistic generative models is seen to minimise future prediction errors (or Bayesian surprise), based on estimates of hidden (i.e., unknown) world states. For instance, when uncertainty in prior expectations is high or environmental volatility increases (e.g., when conditions become more unpredictably-changeable), individuals tend to rely more heavily on incoming sensory feedback and will adjust their visual search strategies accordingly^30,36,37^. Alternatively, when sensory information is more uncertain, more emphasis will be placed on longstanding prior expectations and ‘top-down’ attentional processes^12,38^. Such Bayes-optimal adjustments have been demonstrated in neurotypical cue combination, interceptive timing, movement planning, and visuomotor integration (see review^12^).

Though research is currently limited, recent evidence from the rubber-hand^39^ and size-weight^30^ illusions suggests that autistic people display inflexibilities in active inference. Specifically, when compared to neurotypical controls, autistic participants appear less inclined to adjust visual search and movement initiation kinematics under non-veridical, uncertain task conditions. Importantly, these differences in context-sensitive processing were not accompanied by any generic attenuations in the use of prior expectations^30,39^. Although these results provide clear support for recent hierarchical frameworks of autism^18,32,33^, neither study experimentally manipulated or quantified environmental statistics over time, meaning that causal links must be made with caution. Moreover, movement-related *impairments* were not examined in either lab-based task, limiting their utility in the development of interventions.

The present work examined how movement is dynamically controlled during multi-sensory interceptive actions, where autistic people often display performance impairments^4–7^. To this end, we adopted an immersive virtual racquetball task^14,40^ and monitored how predictive control is adjusted between different volatility conditions. Here, the use of virtual-reality (VR) facilitated systematic, unconstrained manipulations of environmental uncertainty, meaning that we could decipher precisely which predictive processing mechanisms are implicated in autism. Specifically, VR enabled us to artificially alter whether the ‘bounciness’ of an approaching ball remained stable or unpredictably-changeable (*volatile*) over time, before measuring how sensorimotor behaviours were adjusted. Atypical predictive processes usually manifest most clearly in uncertain or volatile conditions, as suboptimal probabilistic expectations will impair abilities to distinguish random sensory noise from actual environmental changes^22,32^. Accordingly, we hypothesised that autistic participants would show impaired interceptive performances, particularly under volatile conditions.

In this task, both the *timing* and *location* of anticipatory eye movements are affected by prior expectations and incoming visual information^14^. Specifically, anticipatory saccades move gaze ahead of the ball to its expected future location, with the subsequent fixation point proving directly proportional to both its early-flight trajectory and, crucially, its predicted elasticity profile^14,40^. These sampling behaviours appear fundamental in the retrieval of post-bounce position information, with unexpected and computationally ‘surprising’ changes in ball bounciness leading to poorer subsequent gaze pursuit^41^. We hypothesised that autistic participants would be less inclined to use a predictive gaze strategy than their neurotypical counterparts, and would thus show later pre-bounce saccades, shorter fixations around the bounce point, and a reduced distinction between expected and unexpected gaze tracking responses (i.e., reduced behavioural surprise to unexpectedly ‘bouncy’ ball trajectories). Furthermore, on the basis that autistic people may be hypersensitive to environmental change^32^, we hypothesised that the ASD group would show greater changes in these measures between stable and volatile task conditions.

Action predictions are also used to guide an individual’s motor response^40,42^. In interceptive skills, swing onset times are flexibly adjusted according to previous ball trajectories and spatiotemporal conditions^40,43^, via precision-mediated sensory attenuation^13,16,17^. Research from constrained motor tasks indicates that movement onset kinematics are suboptimal in autism^5,8,25^, with further scrutiny required in unconstrained movement skills. Moreover, for dynamic and naturalistic actions, the optimal regulation of neuromuscular systems and movement degrees of freedom rests on context-sensitive modulatory mechanisms (e.g., precision control^13^). During uncertain task conditions, for example, neurotypical adults have been shown to increase joint stiffness and restrict multi-effector redundancy^44^. Though such ‘fixing’ of joint angles is less efficient, and would usually be associated with more novice-like movement profiles (i.e., reduced movement degrees of freedom^45^), it likely represents an active attempt to reduce uncertainty from signal-dependent noise^44^. Given that autistic people are proposed to interact with the world as if it is generally uncertain or volatile^32^, we hypothesised that the ASD group would show greater ‘fixing’ of joint angles than the neurotypical group (i.e., reduced range of motion and peak hand displacement: see Table 2 in methods). In line with previous studies^30,39^, these participants were also expected to display inflexible motor kinematics, as evidenced by reduced between-condition adjustments in swing onset time and peak hand velocity.

## Results

**Figure 1:**
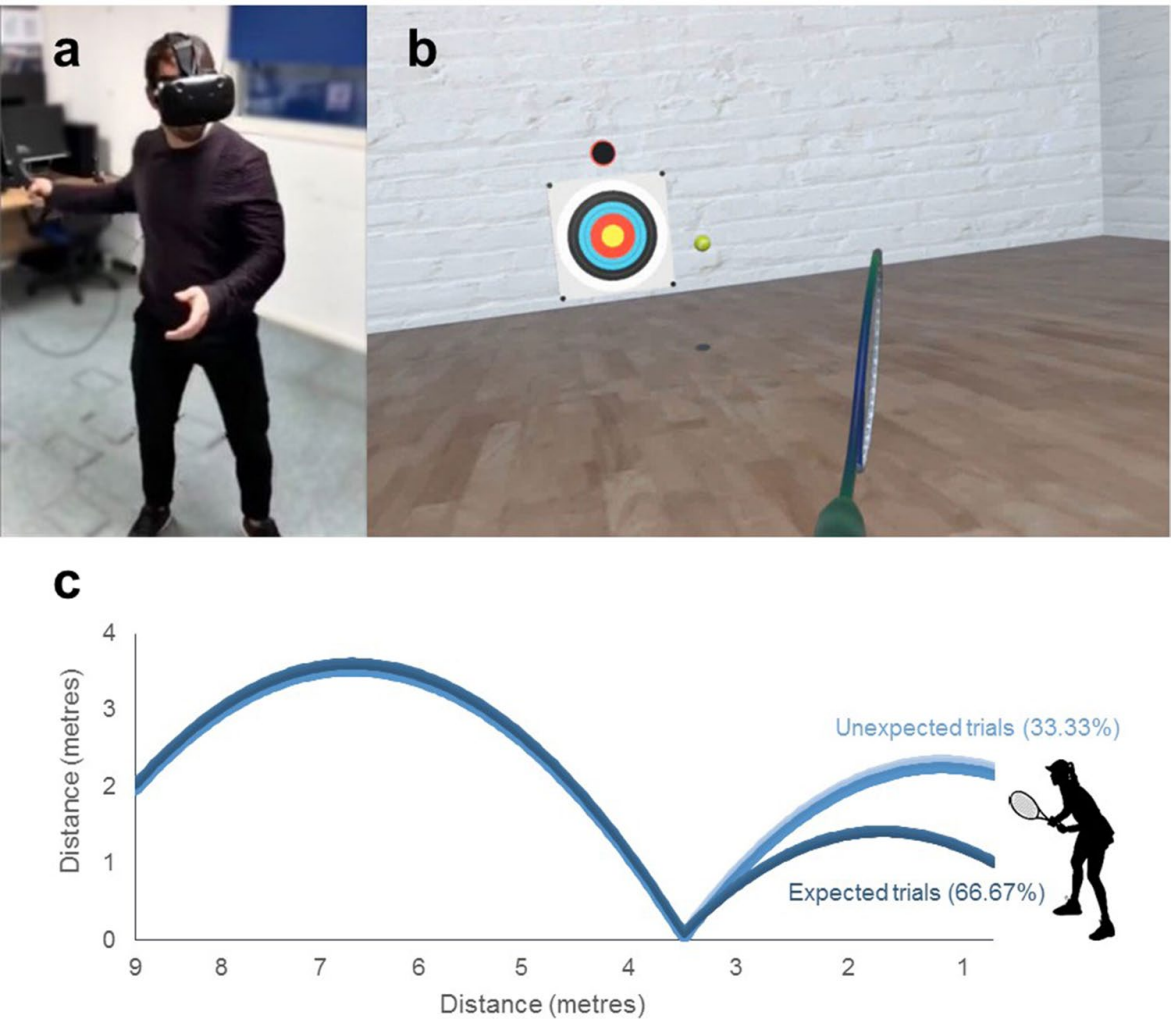
The Virtual Racquetball task. An illustration of the experimental set-up (**a**), example gameplay footage (**b**), and a side-view of ball trajectory distributions (**c**). *Note:* for all trials, virtual balls stayed fixed on the midline of the room and followed the same pre-bounce speed and trajectory. Differences between expected and unexpected trials were therefore consigned to ball elasticity (i.e., ‘bounciness’) manipulations only. See Supplementary Videos of the protocol at https://osf.io/ewnh9/.

An immersive virtual racquetball task required participants to wear an HTC Vive Head-Mounted Display and intercept balls with a controller during two counterbalanced conditions (Fig. 1). On each trial, a single ball bounced toward the participant with either standard (i.e., expected) or unexpectedly-high levels of elasticity. Under *stable* conditions, trials were presented in predictable serial orders, with the probability of facing an expected ball remaining fixed at 67.67%. Under these contexts, Bayes-optimal performers will employ highly ‘predictive’ sensorimotor behaviours^12^, such as early anticipatory saccades, and a clear distinction between expected and unexpected trials. Contrastingly, in the *volatile* condition, the likelihood of facing an expected ball switched unpredictably over time between highly- (83.33%), moderately- (67.67%) and non-predictive (50%). Under this uncertainty, Bayes-optimal performers should increase the sampling of incoming sensory information and show a reduced distinction between expected and unexpected ball tracking behaviours (i.e., reduced behavioural surprise). As both conditions contained the same overall proportion of expected trials (66.67%), general movement and visual sampling strategies could be examined through averaging data retrieved from inbuilt VR hand- and eye-tracking technology. However, to further scrutinise the distinction between expected and unexpected gaze profiles, probability-matched ‘test’ trials were situated within each condition (see methods). These metrics were then compared between autistic (*n* = 26) and neurotypical (*n* = 54) groups.

### Performance data

**Figure 2:**
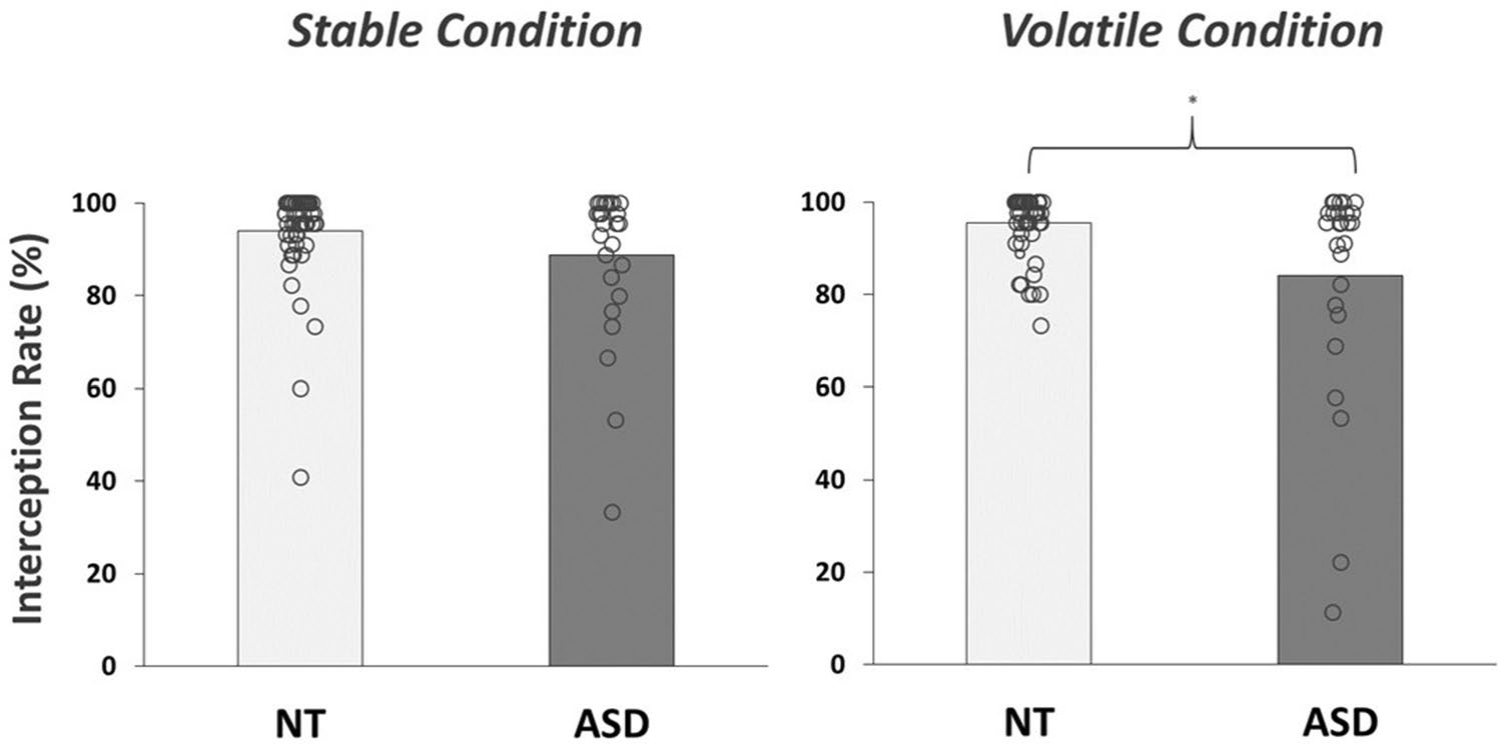
Performance data. The proportion of balls successfully intercepted in stable and volatile conditions for each group. *NT* neurotypical, *ASD* autism spectrum disorder. *denotes statistically significant differences (*p* < .05).

The proportion of successful interceptions revealed a negative skew due to a high number of participants successfully hitting the ball in all trials (*n* = 18; Fig. 2). However, a range of interception rates were still exhibited, particularly in the autism group (range: 27.78–100%). A mixed-model ANOVA showed that performance levels statistically differed between groups (*F*(1,78) = 7.92, *p* = 0.01, *np*^*2*^ = 0.09, BF_10_ = 7.07), with lower interception rates evident in autistic (86.38 ± 19.20%) as opposed to neurotypical participants (94.69 ± 7.19%). These overall scores were not significantly different between stable and volatile trials (*F*(1,78) = 1.13, *p* = 0.29, *np*^*2*^ = 0.01, BF_10_ = 0.18). However, there was a significant condition-by-group interaction (*F*(1,78) = 5.08, *p* = 0.03, *np*^*2*^ = 0.06, BF_10_ = 1.90), with autism-related performance impairments emerging under volatile conditions (Fig. 2; *W* = 963.00, *p* < 0.01, BF_10_ = 21.50). Spearman’s Rho analysis supported these observations, with self-reported autistic-like trait scores across the entire sample (on the 26-item Autistic Quotient: AQ-26) negatively correlating with interception rate in the volatile (*R*_*s*_ = -0.25, *p* = 0.02, BF_10_ = 35.18) but not stable trials (*R*_*s*_ = -0.09, *p* = 0.44, BF_10_ = 1.03). Post-hoc tests indicated that these associations were driven by high-order social and attentional traits (Supplementary Table 1).

### Kinematic data

To examine these performance differences further, we compared aspects of swing kinematics based on the position of the VR hand controllers, between groups and conditions. Three participants (ASD: *n* = 1; NT: *n* = 2) were excluded from this analysis, following detection of univariate outliers or invalid trials (remaining *n* = 77). For peak velocity of the hand, ANOVA showed a significant effect of group (*F*(1,75) = 5.18, *p* = 0.03, *np*^*2*^ = 0.07, BF_10_ = 2.38) but not condition (*F*(1,75) = 0.04, *p* = 0.84, *np*^*2*^ < 0.001, BF_10_ = 0.19), with autistic participants employing slower foreswings than neurotypical individuals (*t*(75) = 2.28, *p* = 0.03, BF_10_ = 2.20). However, the timing of peak velocity was not significantly different between groups (*F*(1,75) = 1.79, *p* = 0.19, *np*^*2*^ = 0.02, BF_10_ = 0.69), and occurred close to ball contact in both conditions (Table 1). Though swing onset times occurred later in volatile trials (F(1,75) = 4.47, *p* = 0.04, *np*^*2*^ = 0.06, BF_10_ = 0.74; Table 1), no group differences emerged (*F*(1,75) = 1.82, *p* = 0.18, *np*^*2*^ = 0.02, BF_10_ = 0.76). Moreover, no significant interactions or correlations were present for these swing onset and peak velocity variables (*p*’s > 0.23; all BF_10_ < 0.50), except for peak hand velocity, which was inversely related to AQ-26 scores (*R* = -0.25, *p* = 0.03, BF_10_ = 1.59). Therefore, autistic participants exhibited slow, novice-like^46^ movement kinematics in both task conditions.

**Table 1:** Foreswing Kinematic Averages (SD) during each Experimental Condition.

	NT group		ASD group	
	Stable	Volatile	Stable	Volatile
**Kinematic measures**				
Swing onset time^**#**^	0.59 (0.10)	0.60 (0.10)	0.55 (0.10)	0.57 (0.08)
Peak velocity of the hand*	10.15 (2.93)	9.96 (3.01)	8.41 (2.79)	8.54 (2.98)
Time of peak hand velocity	-0.04 (0.02)	-0.04 (0.03)	-0.04 (0.02)	-0.04 (0.02)
Maximum hand displacement*	0.61 (0.06)	0.61 (0.07)	0.55 (0.08)	0.55 (0.07)
Swing range of motion*^,#^	83.06 (25.63)	79.94 (27.24)	67.31 (28.01)	65.31 (31.10)

*ASD* autism spectrum disorder, *NT* neurotypical.

*Significantly different between groups (*p* < .05); ^**#**^significant differences between conditions (*p* < .05).

During the foreswing action, autistic participants kept their hands closer to the body (Maximum Hand Displacement: *F*(1,75) = 13.84, *p* < 0.001, *np*^*2*^ = 0.16, BF_10_ = 55.09) and employed reduced ranges of motion (swing ROM; *F*(1,75) = 5.35, *p* = 0.02, *np*^*2*^ = 0.07, BF_10_ = 1.65) compared to their neurotypical counterparts. For swing ROM, a weak main effect for condition also emerged (*F*(1,75) = 4.08, *p* = 0.047, *np*^*2*^ = 0.05, BF_10_ = 1.94), with average values in both groups reducing between stable and volatile trials (*t*(76) = 2.33, *p* = 0.02, BF_10_ = 1.60). The condition-by-group interaction, however, was not significant (*F*(1,75) = 0.19, *p* = 0.66, *np*^*2*^ = 0.003, BF_10_ = 0.37), with volatility-related changes in swing ROM proving similar between groups. Therefore, autistic participants showed higher, more uncertain-like swing ROM values in *both* stable and volatile conditions (Table 1). Relatedly, lower movement degrees of freedom were associated with higher AQ-26 scores across the whole sample, both for maximum hand displacement (*R* = -0.37, *p* = 0.001, BF_10_ = 27.10) and swing ROM (*R* = -0.24, *p* = 0.03, BF_10_ = 1.33). Nonetheless, changes in swing ROM were highly variable (ΔROM range: -31.17 – 24.08, SD: 10.36°), and there was a lack of condition-related changes in maximum hand displacement (*F*(1,75) = 0.07, *p* = 0.79, *np*^*2*^ = 0.001, BF_10_ = 0.20). Therefore, the extent to which these more novice-like joint motions reflected aberrant volatility processing was unclear, and precise examination into participant’s ‘active’ sensory sampling behaviours were required.

### Gaze data

**Figure 3:**
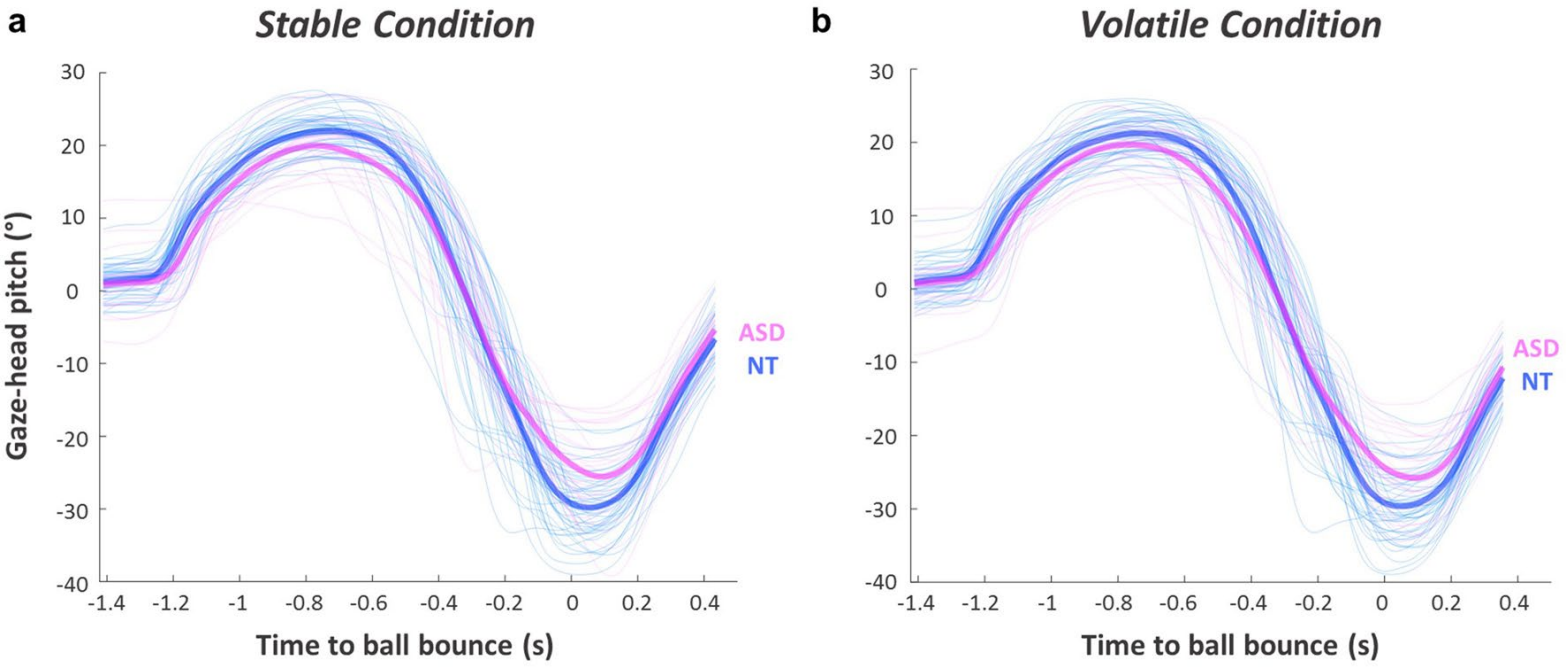
Gaze strategies during the virtual racquetball task. Average pitch of the gaze-in-world vector during stable (**a**) and volatile (**b**) conditions. Pitch represents the vertical angle of a vector which originates from the head at eye-height. Values of zero represent a vector that is parallel to the floor plane, while more positive values indicate that an individual is looking relatively higher in space around the bounce point. Bold lines are group averages, thin lines denote individual cases. *NT* neurotypical, *ASD* autism spectrum disorder.

**Figure 4:**
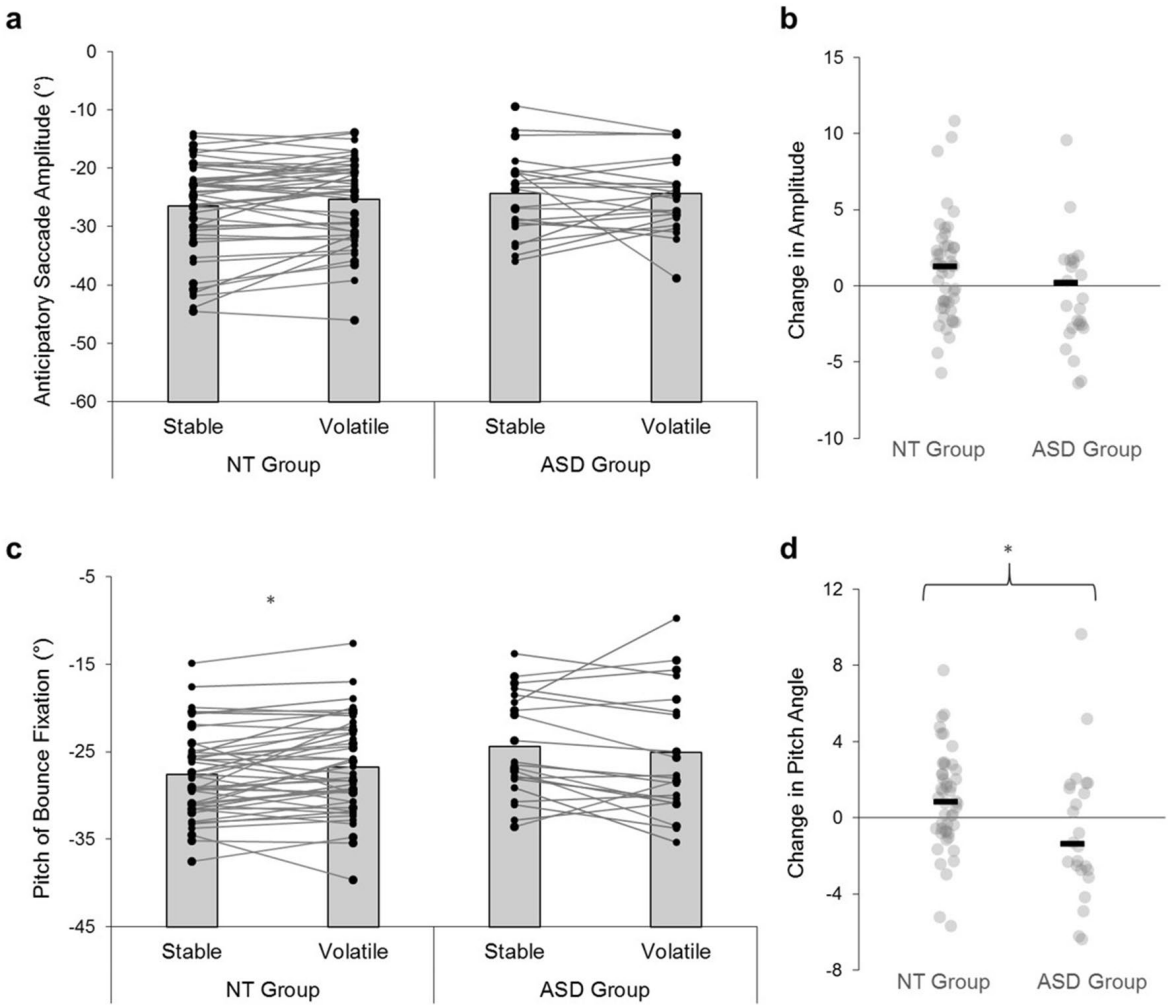
Adjustments in predictive gaze positions. The amplitude of anticipatory pre-bounce saccades (**a**) and subsequent gaze fixation locations (**c**) during stable and volatile conditions. Values represent angular coordinates of the gaze-in-world vector (°), with between-condition changes illustrated in (**b**) and (**d**). *NT* neurotypical, *ASD* autism spectrum disorder; * denotes statistically significant differences (*p* < .05).

Eye-tracking data from eight participants (ASD: *n* = 2; NT: *n* = 6) were identified as poor quality and were excluded from gaze analyses (remaining *n* = 72). As described previously^14,40^, participants utilised a prediction-driven gaze strategy (illustrated in Fig. 3). Specifically, after pursuing its early-flight trajectory, gaze tended to shift ‘predictively’ ahead of the ball via large, anticipatory *pre-bounce saccades*. Gaze then stayed relatively still and focused on a location just above the ball’s future bounce position, in what is referred to hereafter as the *bounce fixation* location. This fixation was generally maintained for ~ 200 ms (mean: 182.16 ± 63.28 ms) until the ball ‘caught up’; when participants would attempt to track the ball onto the racquet through a combination of smooth pursuit and corrective saccades. Interestingly, these general strategies were favoured by *all* participants, irrespective of their diagnosis status (Fig. 3). ANOVAs showed that the timing and amplitude of participants’ pre-bounce saccades were not affected by condition or group (*p*’s > 0.29; all BF_10_ < 1; Fig. 4a, b), nor were they correlated with AQ-26 scores (*p*’s > 0.24; BF_10_ < 0.33). Moreover, the duration of the subsequent bounce fixation was not significantly affected by volatility, diagnosis status, or levels of autistic-like traits (*p*’s > 0.06; BF_10_ < 1.07). Therefore, anticipatory gaze adjustments were evident in both groups, and these prediction-driven responses proved robust to changing environmental conditions.

Notably, both groups attempted to closely pursue balls after they had bounced on each trial (Fig. 3). These tracking behaviours would presumably be impaired if any oculomotor deficits were present. As such, we studied the vertical distance between participant’s gaze and the centre of the virtual ball on a frame-by-frame basis (in angular pitch coordinates), and averaged these values for the post-bounce portion of each trial. Here, greater deviation values would reflect larger average distances between gaze and ball vectors (i.e., high tracking error^42^). However, ANOVA showed no significant main effects (condition: *F*(1,70) = 0.16, *p* = 0.69, *np*^*2*^ = 0.002, BF_10_ = 0.18; group: *F*(1,70) = 3.63, *p* = 0.06, *np*^*2*^ = 0.05, BF_10_ = 1.36) or interactions (*F*(1,70) = 0.66, *p* = 0.42, *np*^*2*^ = 0.01, BF_10_ = 0.43) for this measure. Moreover, these gaze tracking profiles were unrelated to AQ scores (*R* = 0.19; *p* = 0.11; BF_10_ = 0.50) and null group differences emerged when inspecting high-elasticity trials *only* (i.e., trials which had higher, faster-moving balls; see Supplementary Analyses). On this basis, it seems unlikely that sensorimotor difficulties were driven by any generic motion tracking deficits or oculomotor impairments in this task.

However, potential differences in the *position* of participant’s final pre-bounce gaze fixation appeared (see time ‘0’ in Fig. 3). Typically, people will look higher above the floor when they are expecting more ‘bouncy’ ball trajectories^14,40^. Though anecdotal group differences (BF_10_ = 1.32) did not reach significance for this variable (*F*(1,70) = 3.47, *p* = 0.07, *np*^*2*^ = 0.05), there was a significant group-by-condition interaction which required inspection (*F*(1,70) = 4.72, *p* = 0.03, *np*^*2*^ = 0.06, BF_10_ = 1.51). Bounce fixations were higher in autistic than neurotypical participants, but only in stable trials (stable: *t*(70) = 2.59, *p* = 0.01, BF_10_ = 4.08; volatile: *t*(70) = 1.12, *p* = 0.27, BF_10_ = 0.44). These context-sensitive effects were caused by volatility-related increases in the neurotypical group (*t*(47) = 2.42, *p* = 0.02, BF_10_ = 2.16; Fig. 4c, d), who adjusted their fixations more readily under volatile conditions to facilitate the pursuit of ‘bouncier’ ball trajectories (see Supplementary Fig. S6). Autistic participants did not show between-condition changes in this manner (*t*(23) = 0.96, *p* = 0.35, BF_10_ = 0.32, Fig. 4c, d), and instead showed a generally elevated gaze profile around the point of bounce (Fig. 3). They also appeared to update their bounce fixation location more variably on a trial-by-trial basis (Supplementary Fig. S6). Therefore, as with their swing kinematics (ROM: Table 1), autistic participants appeared to display behaviours that are typically affiliated with more uncertain conditions. However, the pitch angle of bounce fixations was unrelated to AQ-26 scores across the whole sample (*p*’s > 0.13; BF_10_ < 0.50), and our weak anecdotal evidence against the null must be interpreted with caution (BF_10_ = 1.51). Further scrutiny into the relationship between autism, prediction, and context-sensitive gaze control is thus required.

Finally, we also distinguished gaze tracking responses between *expected* and *unexpected* trials. As described above, positional distances between the gaze and ball vectors were averaged in the vertical plane for the post-bounce portion of each trial. In this case, we specifically focused on probability-matched ‘test’ trials and subtracted normalised expected (E) values from their unexpected (UE) trial equivalents (see methods). The resulting UE-E difference scores indexed levels of ‘surprise’ to unexpected events. Higher scores would signal that participants were tracking expected balls more closely than unexpectedly bouncy ones (i.e., with less error). Conversely, values close to zero would indicate minimal differences between ball tracking responses. Two participants were excluded from this analysis due to missing data on ‘test’ comparison trials (remaining *n* = 70).

**Figure 5:**
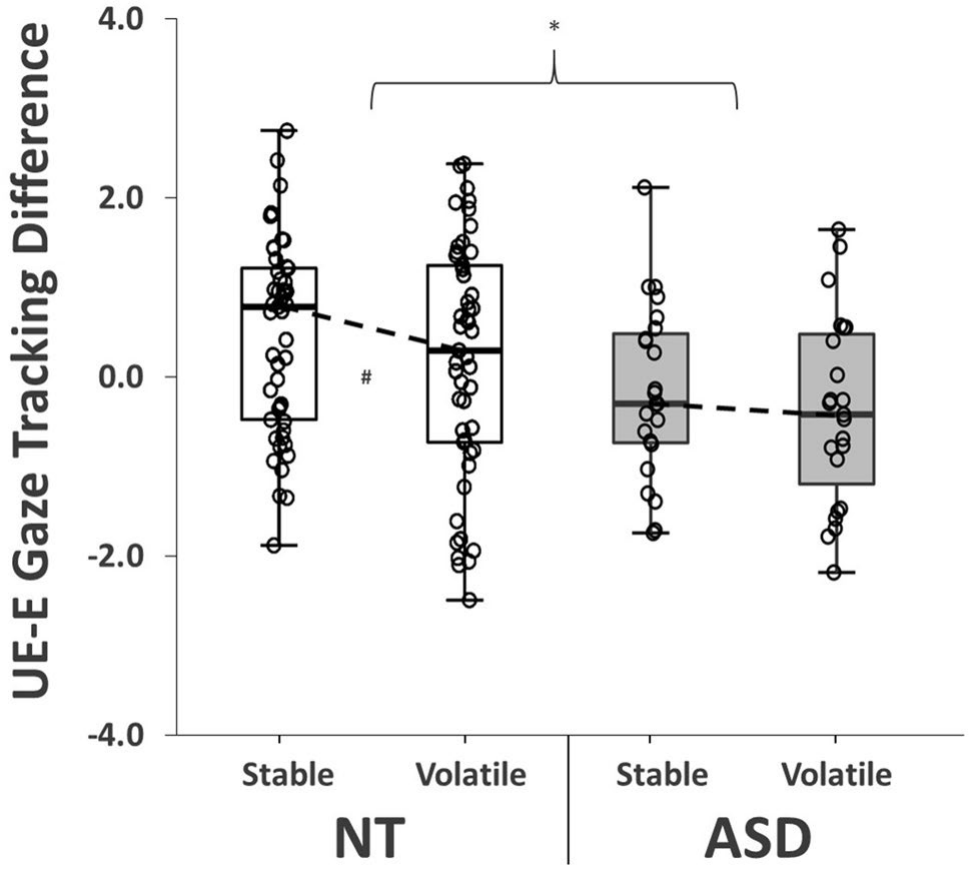
Gaze Tracking Responses. Group differences in gaze tracking behaviours between expected (E) and unexpected (UE) test trials. Higher index values signify more ‘prediction-driven’ errors in post-bounce gaze pursuit (i.e., greater behavioural surprise when faced with the unexpectedly ‘bouncy’ balls). *NT* neurotypical, *ASD* autism spectrum disorder. *denotes significant between-group difference (*p* < .05); ^#^denotes significant change between conditions (*p* < .05).

Manipulation checks indicated that UE-E gaze tracking difference scores were significantly greater than zero under stable conditions (*t*(69) = 2.61, *p* = 0.01, BF_10_ = 2.98). Unsurprisingly, participants tracked expected balls more closely than unexpected ones for these trials. ANOVA revealed a significant effect of condition on this index of behavioural ‘surprise’ (*F*(1,68) = 6.38, *p* = 0.01, *np*^*2*^ = 0.09, BF_10_ = 4.37), with UE-E differences decreasing under volatile conditions (*t*(69) = 2.67, *p* = 0.01, BF_10_ = 3.46). Crucially, there was a significant effect of group on these scores (*F*(1,68) = 5.80, *p* = 0.02, *np*^*2*^ = 0.08, BF_10_ = 3.22). When compared to neurotypical individuals, autistic participants showed generally reduced surprise– they were tracking unexpectedly bouncy balls with a similar level of accuracy to the more expected ones (Fig. 5). This is despite these balls having higher post-bounce velocities and lower prior probabilities. Furthermore, there were also significant negative relationships between UE-E differences, AQ-26 scores (*R* = -0.25, *p* = 0.04, BF_10_ = 1.19) and interception rates (*R*_*s*_ = 0.30, *p* = 0.01, BF_10_ = 3.46). However, no interaction effects emerged for this metric (*F*(1,68) = 0.01, *p* = 0.92, *np*^*2*^ < 0.001, BF_10_ = 0.27), illustrating that autistic individuals adapted sampling behaviours ‘typically’ between conditions.

## Discussion

We examined how sensorimotor control is dynamically adjusted in autism, using a novel and immersive VR paradigm which systematically varied environmental volatility over time. Here, the frequency with which participants successfully intercepted a virtual bouncing ball was significantly lower in autistic individuals (Fig. 2), confirming basic impairments in interceptive skill execution^4–7^. Such performance difficulties were accompanied by atypical swing kinematics (Table 1), gaze fixation patterns (Figs. 3, 4), and levels of behavioural surprise (Fig. 5). Results therefore support active inference formulations of predictive processing^18^, and suggest that the dynamic regulation of sensory sampling and motor control behaviours is fundamentally different in autistic people.

In accordance with predictive processing theories^22,33^, autism-related difficulties in sensorimotor performance were more pronounced under volatile conditions (Fig. 2). Such results align with findings from more constrained prediction-based tasks (e.g., statistical learning^34^ and sensorimotor illusion^39^ paradigms), where autism-related atypicalities emerge under inconsistent, uncertain or unstable probabilistic conditions (see also recent review^28^). Furthermore, the observed differences in this task appeared specific to ASD, and were not a result of any singular subset of autistic-like traits (see Supplementary Table S1 & Fig. S1–S5) or confounding clinically-diagnosed conditions (e.g., identified co-occurring motor disorders). Therefore, our novel, systematic assessment of sensorimotor control extends our neuro-computational understanding of autism into more dynamic and unconstrained environments, where optimal behaviours rest on hierarchical, iterative predictive processing.

Autistic participants employed arm swing actions that were lower in peak velocity, closer to the body, and more restricted in ROM (Table 1). These profiles are indicative of more novice-like swing mechanics, as actions are typically slower and have reduced degrees of freedom in the early stages of learning^45,46^, and autism-related sensorimotor difficulties may thus reside at the kinematic level^7,9,10^. Indeed, atypical peak hand velocities have been consistently reported in clinical visuomotor research^9,47,48^ and could result from various central and/or peripheral factors, including aberrant predictive action modelling^9^. However, contrary to our hypotheses and previous studies^5,8^, kinematic group differences were not significant for any of our movement initiation metrics (Table 1). Therefore, when examined in isolation, it is unclear whether these atypical movement kinematics in autism reflect specific differences in predictive processing, or more general impairments in sensorimotor development.

We next sought to explore the precise mechanisms that drive these motor differences. Notably, participants’ gaze kinetics were remarkably robust to the highly-changeable probabilistic conditions (Fig. 3), which reinforces suggestions that a prediction-driven visual sampling strategy is optimal for dealing with dynamic and uncertain cues for this type of task^14,42^. Our data shows that autistic individuals also employed this ‘top-down’ strategy (Fig. 3) and that they shifted their visual attention and tracked approaching balls similarly to neurotypical individuals (see Supplementary Analyses). These findings undermine proposals of broad attentional differences^49^ and/or generic attenuations in the use of prior knowledge^19^. Moreover, null group differences were observed in relation to participants’ anticipatory pre-bounce saccades, despite these eye movements being directly related to previous trial trajectories and task constraints^14,40,43^. Consequently, our results join varied evidence against simple Bayesian theories of autism, and support conclusions that predictive processing abilities are *not* generically impaired in sensorimotor tasks^28–30^.

Recent research suggests that sensorimotor difficulties in autism may instead stem from context-sensitive mechanisms relating to precision modulation and volatility processing^18,32,33^. In this study, participants displayed subtle adjustments in visual sampling behaviour that were qualitatively consistent with optimal active inference. Specifically, when conditions were more uncertain, individuals appeared to rely *less* on prior information and *more* on incoming, stimulus-driven attentional cues^36,37^. This was illustrated in our gaze data, where the tendency to track expected balls more closely than unexpected ones was reduced under volatile conditions (Fig. 5). Participants also adjusted their fixations more variably in these trials (Supplementary Fig. S6). Such context-sensitive patterns of data match results from psychophysics experiments, where unexpected cues are processed more rapidly under uncertain conditions e.g.,^50^. The changes observed here reflect volatility-related modulation of precision and learning rate, which increases an individual’s responsivity to salient events^15^.

Strikingly, our data showed consistent autism-related atypicalities in this context-sensitive modulation of sensorimotor control. Indeed, autistic participants showed differences in swing ROM (Table 1), bounce fixation location (Figs. 3, 4; Supplementary Fig. S6), and behavioural surprise (Fig. 5); metrics which all appeared sensitive to volatility conditions. For each of these measures, the ASD group demonstrated behaviours that are typically associated with high environmental instability. For instance, differences in gaze tracking between expected and unexpected ‘test’ trials were significantly reduced in autistic participants (Fig. 5), indicating dampened surprise to unexpected events (as in recent neurological and behavioural evidence^24,32^). Similarly, while neurotypical participants reduced swing ROM during volatile conditions *only*, autistic participants exhibited low ROM scores across both conditions (Table 1). These atypical movement profiles can be explained by an increased tendency to ‘expect the unexpected’ in autism^32^, as a greater fixing of joint angles may serve as an active attempt at reducing uncertainty (i.e., through reducing signal-dependent motor noise^44^). Therefore, our study supports proposals that autistic people tend to interact with the world like it is highly unstable or uncertain^32^.

Atypical volatility processing can explain difficulties with various activities of daily living in autism, including sensorimotor impairments^18^. In our study, participants who showed poorer task performance and higher autistic-like traits tended to sample the world in a more uncertain-like manner (Supplementary Fig. S5). This is unsurprising, as the majority of balls bounced in an ‘expected’ way, so it would be suboptimal to sample cues as if they are unrelated to long-term prior experience. However, our fixation analysis cautiously suggests that autistic participants predictively positioned their gaze at a higher, more variable location than their neurotypical counterparts (Fig. 3; Supplementary Fig. S6), in a manner that benefits the sampling of recent high-elasticity ball trajectories^14^. Though it is unclear whether these differences resulted from atypical learning rates^32^ or compensatory, non-linear adaptations in gaze behaviour (e.g., ‘centering’ strategies^51^), these results reinforce the notion that autistic participants were overestimating volatility, or ‘expecting the unexpected’, during the task^32^.

Nevertheless, the exact source of aberrant uncertainty expectations and volatility modulation in autism remains to be explored^18,33^. Contrary to our initial hypotheses, autistic and neurotypical groups appeared to comparably adjust visual sampling and motor kinematics according to environmental (in)stability (Table 1; Fig. 5). These null effects are notable, as recent computational models posit that autistic people are *hypersensitive* to environmental change, due to dysfunctions in neural excitation and/or modulation^32,33^. Though conflicting with these proposals, such findings align with reinforcement learning data^52^, which suggest that atypicalities may be consigned to higher-level processing computations. Our task was unlikely to implicate such mechanisms, with visual motion cues about ball-flight dynamics likely occurring in lower hierarchical levels. It is also possible, however, that our ASD group data do *not* highlight atypicalities in volatility processing, but rather a broad, psychobehavioural intolerance of uncertainty. Indeed, behavioural inflexibility and an insistence on sameness are well-defined autistic-like traits that correlate with motor difficulties^11^. While these traits have been conceptually linked to predictive processing atypicalities^22,33^, statistical associations do not consistently materialise^29^. Therefore, research must establish whether sensorimotor difficulties reflect abnormalities in neuromodulation (e.g., in noradrenergic responsivity, divisive normalisation) or secondary consequences of cognitive and behavioural traits.

A number of study limitations must also be considered. For example, we did not directly assess participants’ cognitive or visual abilities, nor were there any checks performed for undiagnosed motor conditions. Such variables could have influenced our data, with autistic populations showing higher incidence rates of cognitive impairment, optometric issues (e.g., strabismus^53^) and developmental disorders^54^. Though participants were excluded if they reported co-occurring medical conditions (see methods), many of these issues can remain undetected. Levels of experience in racquet-based activities were also unclear and may generally be lower in clinical groups^55^. However, our correlational analysis did examine relationships between sensorimotor control and levels of autistic-like traits across a large general population (i.e., the broader autism phenotype^54^). Most participants in this analysis were neurotypical (68%), which reduces the influence of ASD-related confounds^54^. Notably, all but one of the between-group effects that were identified in our main analysis were accompanied by significant AQ correlations (Supplementary Fig. S1–S5). These trait-based effects notably reinforce our main findings, though future research could explore additional co-variables in their analyses (e.g., IQ subscale scores, clinical questionnaires, standardised motor assessments).

Additionally, impoverished depth cues and haptic feedback in our VR task could influence action control and uncertainty expectations^56^, thus limiting their generalisability to ‘real-world’ behaviour. Although this argument is, in itself, uncertain at present^56^, and the use of VR affords unique methodological benefits, future studies may wish to manipulate probabilistic conditions in ‘real-world’ tasks. To do this, one may wish to select a task that is more sensitive to prior expectations. Indeed, though gaze strategies are evidently driven by prediction in our task (Fig. 3)^42^, time-pressed interceptive actions still rely heavily on incoming visual information^14^. Therefore, the addition of prior contextual cues should be considered, such as probabilistic sensory signals (e.g., predictive auditory tones^32^) or explicit prior information^57^. These contextual cues should not only enable research into more predictive control strategies, but they could also form the basis of future sensorimotor interventions (see ‘Moneyball Approach’ in sport^57^).

In conclusion, autistic people tend to struggle with performing an interceptive motor skill when sensory cues are unpredictably changeable over time. These performance difficulties are underpinned by fundamental differences in predictive processing and active inference. Specifically, atypical sensory sampling behaviours and movement kinematics appear driven by aberrant precision modulation and/or volatility processing mechanisms. The exact source of these neuro-computational differences requires further examination.

## Methods

### Participants

Ninety participants visited the laboratory (33 female, 78 right-handed, age: 21.66 ± 4.45 years). Thirty of these individuals had a formal diagnosis of ASD, while the remaining sample (*n* = 60) were age-matched neurotypical individuals (ASD group: 21.40 ± 5.09 years; NT group: 21.78 ± 4.14 years; *t*(88) = 0.70, *p* = 0.70, BF_10_ = 0.25). A large neurotypical sample was recruited to provide sufficient power for correlational analysis (α = 0.05; *1-β* = 0.8; see below). All autistic participants reported that they had received their diagnosis from a qualified clinician according to DSM-IV^58^ or ICD-10^59^ criteria, and completed both the 26-item Autistic Quotient (AQ-26)^60^ and Social Communication Questionnaire (SCQ)^61^ to corroborate clinical presentation of autistic-like traits. Although diagnosis status was not independently verified in this study, a broad range of SCQ scores were displayed by the ASD group that are consistent with normative clinical values^62^ (mean: 18.34 ± 5.72). Participants self-reported normal or corrected-to-normal vision and were excluded if they reported any history of musculoskeletal or neurological disorders, leading to the removal of two cases (ASD: *n* = 1; NT: *n* = 1). Neurotypical participants also completed the AQ-26 (range: 37–80, mean: 55.17 ± 9.77; *n* = 59) to permit correlational analyses across the whole sample (i.e., the broader autism spectrum^54^). All participants were naïve to the experimental aims and had no prior experience of playing VR-based racquet sports. Informed consent was obtained in accordance with British Psychological Society guidelines, and the study received approval from the School of Sport and Health Sciences Ethics Committee (University of Exeter, UK) and Department of Psychology Ethics Committee (University of Bath, UK). The study methods closely followed these approved procedures and the Declaration of Helsinki.

### Apparatus and stimuli

A virtual environment, simulating an indoor racquetball court, was developed using the gaming engine Unity (Unity Technologies, San Francisco, CA). This simulated environment (see Fig. 1) spanned 15 m in length and width, and contained a series of concentric circles projected onto the front wall as an aiming *target*. Above this target was an additional concentric circle, representing the starting location where virtual balls were launched from in each trial (launch height: 2 m). The floor resembled that of a traditional squash court, with participants instructed to start behind the ‘short line’ (located 9 m behind front wall, 0.75 m from the midline; as in Diaz et al*.*^14^). To ensure consistency in this starting position, a 1 m^2^ service box was marked on the laboratory floor with reflective tape, and an experimenter checked that participants were stood in this square prior to all experimental trials.

The virtual environment was presented to participants on an HTC Vive head-mounted display (HTC Inc., Taoyuan City, Taiwan; Fig. 1), a high-precision, consumer-grade VR system which has proven valid for small-area movement research tasks (field of view: 110°, accuracy: 1.5 cm, jitter: 0.5 mm, latency: 22ms^63^). Two ‘lighthouse’ base stations recorded movements of the headset and hand controller at 90 Hz. The headset also included an inbuilt Tobii eye-tracking system, which uses binocular dark pupil tracking to monitor gaze at 120 Hz (spatial accuracy: 0.5–1.1°; latency: 10 ms, headset display resolution: 1440 × 1600 pixels per eye). Gaze was calibrated over five virtual locations prior to each condition, and upon any obvious displacement of the headset during trials.

Participants then attempted to hit balls towards the projected target circles using a virtual racquet (Fig. 1), operated by the Vive hand controller. Virtual balls were 5.7 cm in diameter, and resembled the visual appearance of a ‘real-world’ tennis ball. The visible racquet in VR was 0.6 × 0.3 × 0.01 m, although its physical thickness was exaggerated by 20 cm for the detection of ball-to-racquet collisions (see discussion of tunnelling effects^14,40^). One neurotypical participant was excluded from analyses following frequent loss of headset tracking during their session (remaining *n* = 86).

### Procedures

On arrival to the laboratory, participants provided written informed consent and completed the autistic-like trait questionnaires. Next, they were fitted with the VR headset and presented with a view of the simulated racquetball court. Participants completed six familiarisation trials and the inbuilt VR eye-tracker was subsequently calibrated, before undertaking the *stable* and *volatile* conditions. During each trial, individuals were instructed to hit virtual balls towards the centre of the projected target. Balls were launched from the front wall, following 3 auditory tones, and passed exactly through the room’s midline, bouncing 3.5 m in front of the prescribed starting position. Right-handed participants started 0.75 m to the left of this midline, and left-handers 0.75 to the right of this point, meaning that all shots were forehand swings. Participants were informed that the ball would bounce once, but that they were free to hit the ball before or after it reached them. Task instructions simply stated that they should aim to hit as many balls as possible to the middle of the front target. No further information relating to ball elasticity, trajectory or probabilistic manipulations were provided. Virtual balls followed the same pre-bounce trajectory and speed (vertical speed: -9 m/s at time of bounce; Fig. 1), which were both consistent with the effects of gravity (-9.8 m/s^2^). Although bounces were accompanied by auditory feedback, no visual, proprioceptive, or verbal feedback were provided upon making contact with the ball. Instead, a neutral ‘pop’ sound was incorporated, so as to minimise the influence of motivation and communicative requirements.

To manipulate environmental volatility in each condition, we systematically varied ball elasticity over time (Fig. 1). Specifically, in *expected* trials, ball elasticity was congruent with its visual ‘tennis ball-like’ appearance, and set at 65%. Conversely, in *unexpected* trials, elasticity was increased to 85%, an abrupt change in ‘bounciness’ that is easily detectible to participants^14^. By selecting such unnatural ball elasticity profiles, and by adjusting these without the participant’s knowledge^14,41^, it was anticipated that post-bounce ball trajectory would deviate substantially from any ‘real-world’ prior distributions. This would then permit unique control over participant’s experience of expected and unexpected events, through probabilistically contrasting order sequences (available at https://osf.io/ewnh9/).

Specifically, in *stable* conditions, balls were presented in ‘predictable’ serial orders (e.g., three unexpectedly-bouncy balls would follow three expected ones, and so on), with the likelihood of facing a ‘normal’ ball (i.e., expected event) remaining fixed at 67.67%. In the *volatile* condition, these ball probabilities were unstable, switching irregularly between highly- (83%), moderately- (67%) and non-predictive (50%) in blocks of 6, 9 or 12 trials. Importantly, conditions contained the same number of Expected (*n* = 30) and Unexpected (*n* = 15) trials in ‘high-interference, non-repeating’ schedules^64^, meaning that the difference between blocks was consigned to environmental volatility only (i.e., differences in how labile the context is perceived to be). Furthermore, to permit precise within- and between-condition comparisons, three expected and three unexpected “test” trials were situated within each block. These trials had identical prior probability distributions (66.67% of preceding trials contained expected ball trajectories) and identical previous trial histories (*n –* 1 were all expected trials). To ensure that bouncy balls remained computationally surprising in the stable condition, unexpected ‘test’ trials were taken from within the final nine trials, in which the order sequences had recently been changed.

The experiment began with a practice set of six trials, whereby balls were projected from the target without a bounce (so that ball elasticity remained unknown to participants). Thereafter, upon calibration of the eye-tracking system, experimental conditions were performed in a counterbalanced order. Each condition contained 45 trials and was separated by a short break, with a total of 96 trials performed by each participant.

### Data analysis

To index task performance, the proportion of trials in which participants made contact between the ball and racquet (*interception rate*, %) were recorded. Thereafter, positional data for the hand controller were extracted from the Vive system, and smoothed using a dual-pass, zero-phase Butterworth filter (at 10 Hz^65^). The contact point between the racquet and ball (referred to as: *ball contact frame*) were derived from the last data point before ball exhibited an abrupt change in direction of its trajectory. Trials where participants missed the ball were also included in analyses. In these instances, the reference *ball contact frame* represented the last data point in which the ball’s depth position exceeded that of the racquet. Trials where participants used a backhand swing, as opposed to a forehand swing, were noted at the time of data collection and removed from kinematic analysis.

To capture aspects of swing kinematics, we calculated a number of measures linked to motor proficiency (all defined in Table 2), namely: swing onset time, peak velocity of the hand, maximum hand displacement from the head, and swing Range of Motion (ROM). Specifically, swing onset time was defined from the first frame at which forward motion of the racquet began, while swing offset corresponded with the ball contact frame. The foreswing, representing the forward phase of the hand movement before ball contact^66^, was defined between swing onset and swing offset. Velocity of the hand controller was calculated as the square root of the sum of squared vector differentials^9^, where peak velocity and the timepoint of peak velocity (ms, relative to ball contact frame) was identified during the foreswing phase. Higher peak velocities, which occur close to ball contact, are indicative of more proficient motor control^46^. Normalised maximum hand displacement from the headset denoted the span of the arm from the body during the swing. This was operationally defined as the distance between the headset and hand controller position in the transverse plane (divided by body height in meters). Swing ROM (°) was calculated as the angular deviation of the hand controller during the foreswing. Angular deviation was defined in the transverse plane, with angles of 0° representing minimal rotation. Reductions in maximum hand displacement and/or swing ROM values would signal greater ‘fixing’ of movement degrees of freedom^67^, a motor strategy which could be used to reduce action uncertainty^44^.

**Table 2:** Description of kinematic outcome measures.

Variable	General description	Operationalised definition
Swing onset time	Moment when the racquet first started moving towards the ball	The first timeframe in which forward motion of the VR hand controller was detected (expressed relative to ball contact frame)
Peak velocity of the hand	The highest speed that the hand reached when moving towards the ball	The maximum differential position of the VR hand controller shown between frames following swing onset (expressed in m/s)
Time of peak hand velocity	The moment when the hand reached its highest speed	The time at which Peak Velocity of the Hand occurred, relative to ball contact frame
Maximum hand displacement	The furthest distance that the hand deviated away from the body during the swing action	The maximum distance that occurred between the VR headset and hand controller in the transverse plane following swing onset (normalised by participant body height)
Swing range of motion	The total arc travelled around the body by the hand during the swing action	The total angular deviation (°) of the hand controller from the VR headset that occurred in the transverse plane following swing onset

A single unit vector corresponding to cyclopean gaze direction was extracted from the inbuilt eye-tracking system, with features defined according to head-centred, egocentric coordinates (i.e., vertical and horizontal coordinates). Both this extracted gaze vector, and the ball’s head-centric position were then plotted with respect to 2D direction in space, to provide relative ‘in-world’ angular orientation metrics (see *gaze-head* and *gaze-ball* angles in Table 3). Here, yaw angles represented rotation about a vertical axis that is in-line with gravity, and *pitch* values index angular deviance from a plane originating at eye-height that is parallel to the floor plane^14,40^. All trials were segmented from the moment of ball release until the time point corresponding to ball contact frame. Gaze values were passed through a three-frame median filter, before being smoothed by a second-order, zero-lag Butterworth filter^68^. In line with recent recommendations^43,69^, different cut-off frequencies were applied for saccade identification (50 Hz) and analysis of positional tracking features (15 Hz). Trials with > 20% missing data, or where eye-tracking was temporarily lost (> 100 ms) were excluded.

**Table 3:** Description of gaze metrics.

Variable	General description	Operationalised definition
Gaze-head angle	Where gaze was being directed in space, relative to the head	Angular orientation of the gaze vector in 2D space, with respect to the VR headsets ‘in-world’ position (expressed as pitch and yaw, °)
Gaze-ball angle	Where gaze was being directed in space, relative to the ball	Angular deviation in 2D space between the gaze vector and the ball’s head-centric spatial position (expressed as pitch and yaw, °)
Anticipatory pre-bounce saccade onset time	The moment when gaze suddenly shifted ahead of the ball before it bounces	The median onset time of participants’ final pre-bounce saccadic eye movement (recorded in ms, relative to when the ball had bounced)
Anticipatory pre-bounce saccade amplitude	How far gaze moved when it was being suddenly shifted ahead of the ball (see above)	The change in gaze-head pitch angle (°) that occurred between the onset and offset of participants’ final pre-bounce saccade
Bounce fixation duration	How long gaze remained steady for, around the time when the ball was bouncing	The average duration of gaze fixations that occurred at the time of, or immediately prior to, the ball bouncing on a trial (expressed in ms)
Bounce fixation location	Where gaze was directed around the time when the ball was bouncing	The average gaze-head pitch angle (°) of fixations that occurred at the time of, or immediately prior to, the ball bouncing
Average post-bounce gaze tracking error	How much higher or lower gaze was from the ball, on average, from when it bounced to when it was hit/missed by the racquet	The average gaze-ball pitch angle (°) shown from the first timeframe after the ball bounces up to the point of racquet-ball contact
UE-E gaze tracking difference	How much closer gaze was tracking expected as opposed to unexpected balls after they had bounced	Differences in normalised post-bounce gaze tracking error (see above) between expected and unexpected ‘test’ comparison trials

Angular velocities (°/s) and accelerations (°/s^2^) of gaze-in-world vectors were calculated from the distance between samples of the filtered signal. Saccades were identified from portions of data where gaze acceleration was more than five times its median absolute acceleration^40^. To avoid erroneous detections (e.g., due to pursuit or tracker-noise artefacts), gaze velocity had to exceed 40°/s for five consecutive frames and had to be at least 20% greater than that of the ball, with time periods preceded or followed by missing data also excluded. If this acceleration criteria failed to identify any anticipatory pre-bounce saccades, trials were manually inspected using a 30°/s velocity threshold^43^. Onset and offset times were determined from these signals using acceleration minima and maxima^68^. A spatial dispersion algorithm was then used to extract gaze fixations^70^. Here, fixations were defined from portions of data where gaze velocity was < 30°/s^14^, using a 3° spatial dispersion threshold and a minimum required duration of 100 ms^71^. This method excluded dynamic phases of smooth pursuit and instead highlighted periods in which gaze became stable within a 3° circular area.

Upon identification of saccades and fixation periods, various prediction-related gaze metrics were calculated (described in Table 3). As we were interested in the final predictive saccade made before the ball had bounced, the latency (i.e., median onset time, relative to bounce; ms) and amplitude (i.e., mean deviance between the final and initial gaze position) of this gaze event were recorded. Moreover, we extracted the fixation position at the moment of bounce (expressed as gaze-head pitch angle), in addition to the average gaze-ball pitch after this timepoint. To assess the degree of gaze tracking prediction error, average gaze-ball pitch was converted into z-scores for each participant, with mean expected test scores subtracted from their corresponding unexpected test trial values. This presented a *UE-E gaze tracking* difference score, whereby higher scores would signal a greater difference between expected and unexpected trials (i.e., greater behavioural surprise following an unexpected trial event).

Gaze and kinematic data values that were > 3.29 SD away from the mean were classed as univariate outliers (*p* < 0.001) and removed from analysis (see guidelines^72^). Participants with > 20% of data identified as missing and/or outliers were excluded (*n* = 6). One performance outlier was excluded from analysis, after they failed to intercept the ball on any trials and showed extreme gaze values, potentially due to equipment error and/or a lack of task understanding. Following this case removal, a further two autistic participants were then identified as potential performance outliers (see Fig. 2). However, since the overall pattern of results was not affected by their inclusion, and such extreme values are consistent with previously documented clinical sensorimotor impairments, these cases remained in the analysis (as recommended in clinical guidelines^73^). Remaining missing data points within the dataset (*n* = 80) were deemed missing completely at random, on the basis of Little’s MCAR test (*p* > 0.05). For all variables, normality, linearity, multicollinearity, and homoscedasticity of data were inspected. Cleaned data were analysed using JASP (version 0.12.2), with significance accepted at *p* < 0.05 and data presented ± SD.

Mixed-model ANOVAs assessed the effects of group and condition on all of our metrics relating to performance (interception rate), action kinematics (swing onset time, peak hand velocity, time to peak velocity, maximum hand displacement, ROM) and gaze behaviour (predictive saccade onset time/amplitude, bounce fixation duration/position, average post-bounce gaze tracking error, UE-E gaze tracking difference scores). Any significant differences were examined using two-tailed t-tests and all effect sizes were calculated using partial-eta squared (ηp^2^). To explore the role of autistic-like traits, Pearson’s Correlation analysis explored relationships between all sensorimotor outcomes and AQ-26 scores. As data for interception rate and predictive saccade outcomes violated assumptions of normality, these outcomes were inspected using non-parametric *t*-test and correlation equivalents (i.e., Mann–Whitney U for group comparisons, Spearman’s Rho for correlation analyses). Mixed-model ANOVAs are robust to moderate deviations from statistical normality^74^, and were still performed for these measures. Non-spherical data were adjusted using the Greenhouse–Geisser correction, and multiple comparisons were accounted for using the Holm-Bonferroni method^75^. For all tests, Bayes Factors using a symmetric Cauchy prior quantified the strength of evidence for the alternative and null hypotheses.

## Supplementary information

Supplementary Information.

## Data Availability

All data generated and analysed during the current study are publicly available at: https://osf.io/ewnh9/.
