# Supplementary material for: An examination of active inference in autistic adults using immersive virtual reality
Source: Sci Rep. 2021 Oct 13;11:20377. doi: 10.1038/s41598-021-99864-y (PMC8514518; doi:10.1038/s41598-021-99864-y)
Supplement: Supplementary file 1 — Supplementary Information. [file 41598_2021_99864_MOESM1_ESM.pdf]

Supplementary Information

An examination of active inference in autistic adults using immersive virtual reality.

Tom Arthur, David Harris, Gavin Buckingham, Mark Brosnan, Mark Wilson, Genevieve Williams and Sam Vine

1. Correlational Analyses.

Supplementary Table 1. Spearman's Rho Correlations (R_S) between autistic-like traits and task performance for each condition.

	Interception Rate (%)	
AQ-26 Subscale:	*Stable Condition*	*Volatile Condition*
Numbers and Patterns	-.19	-.19
Social Skills	-.05	-.26*
Routines	-.04	-.12
Attention Switching	-.12	-.24*
Imagination	-.02	-.18
Total Score	-.09	-.25*

AQ-26: shortened autistic quotient; * denotes significant relationship ($p < .05$).

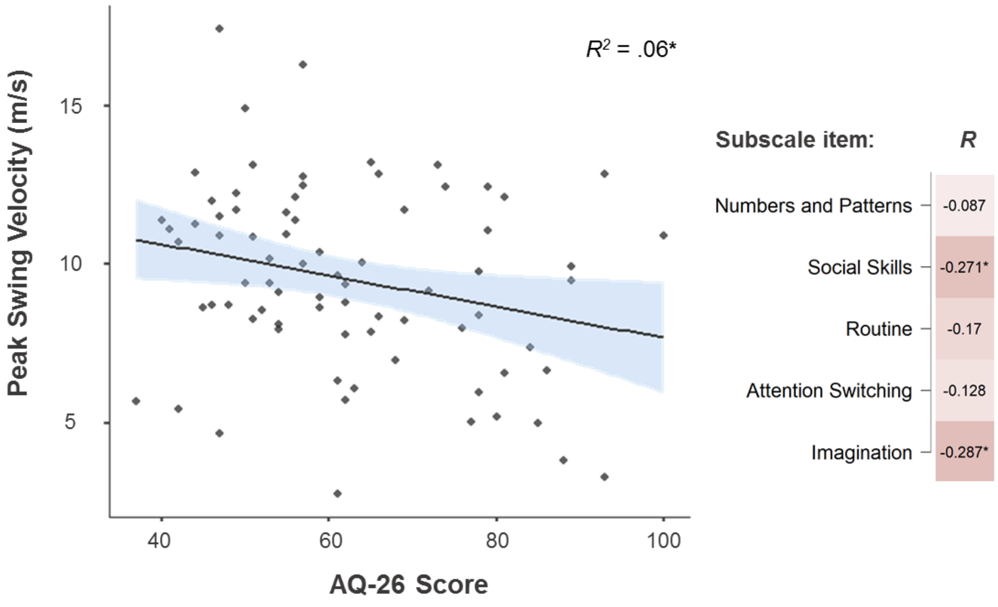

Supplementary Figure S1. Scatterplot showing the relationship between average peak velocity (m/s) and self-reported 26-item autistic quotient (AQ-26) scores. Presented alongside Pearson's Correlation Analysis with each individual subscale. Shaded bars represent standard error of the regression coefficient. Asterisks denote significant statistical relationship (*$p < .05$).

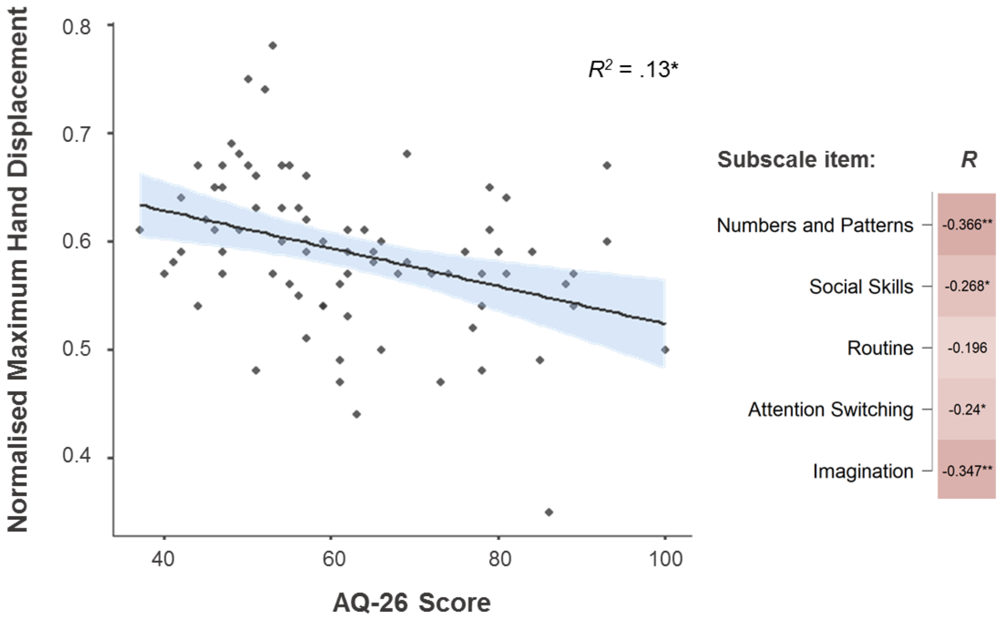

Supplementary Figure S2. Scatterplot showing the relationship between maximum hand displacement values (normalised for participant height) and self-reported 26-item autistic quotient (AQ-26) scores. Presented alongside Pearson's Correlation Analysis with each individual subscale. Shaded bars represent standard error of the regression coefficient. Asterisks denote significant statistical relationships (*$p < .05$; ** $p < .01$).

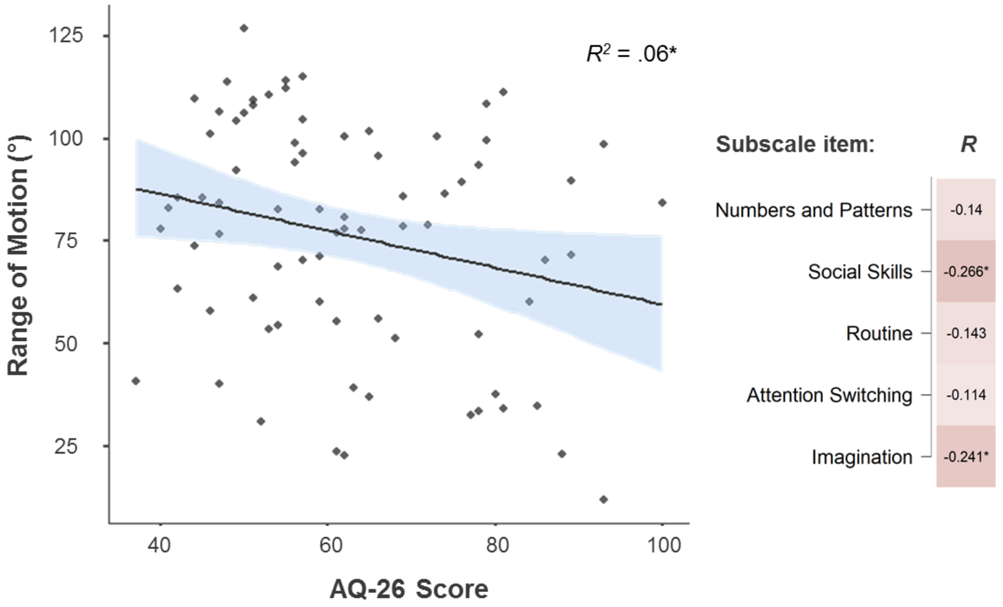

Supplementary Figure S3. Scatterplot showing the relationship between swing range of motion averages and self-reported 26-item autistic quotient (AQ-26) scores. Presented alongside Pearson's Correlation Analysis with each individual subscale. Shaded bars represent standard error of the regression coefficient. Asterisks denote significant statistical relationships (*$p < .05$).

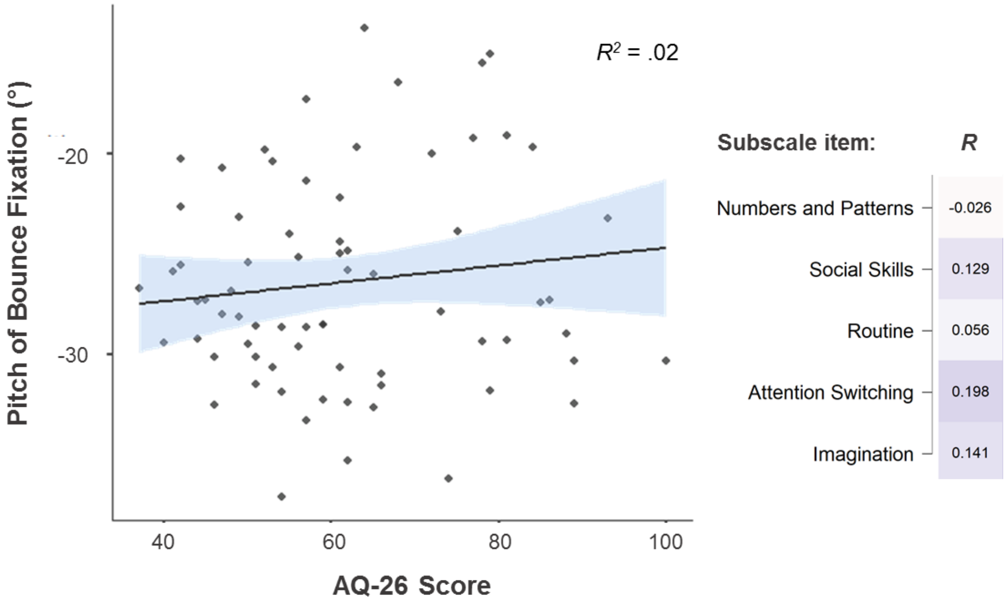

Supplementary Figure S4. Scatterplot showing the relationship between the average spatial location of bounce gaze fixations (pitch angle, °) and self-reported 26-item autistic quotient (AQ-26) scores. Presented alongside Pearson's Correlation Analysis with each individual subscale. Shaded bars represent standard error of the regression coefficient. No significant statistical relationships were observed (*p's* > .05).

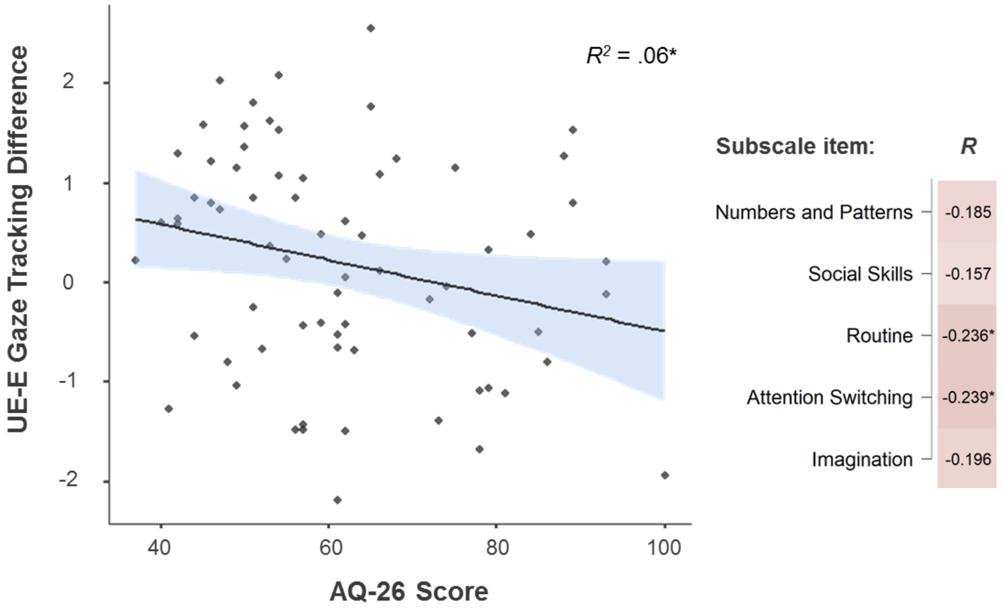

Supplementary Figure S5. Scatterplot showing the relationship between average gaze tracking differences and self-reported 26-item autistic quotient (AQ-26) scores. Presented alongside Pearson's Correlation Analysis with each individual subscale. E: expected test averages; UE: unexpected test averages. Shaded bars represent standard error of the regression coefficient. Asterisks denote significant statistical relationships (*$p < .05$).

2. Post-Hoc Analyses of Gaze Tracking Behaviours

In our main analysis, we found that autistic people use interceptive gaze behaviours that are typically associated with high-uncertainty conditions. While neurotypical participants tended to pursue expected ball trajectories more closely than unexpected ones, autistic individuals appeared to sample both cues with similar levels of accuracy (or 'error'; Fig. 5). These differences were not a result of any significant gaze tracking deficits, nor were they accompanied by any alterations in the *timing* or *amplitude* of key saccadic eye movements (Fig. 3 & 4). Instead, they likely reflect aberrant behavioural surprise computations (see discussion). However, it is possible that atypical gaze responses in ASD stem from underlying attentional and/or oculomotor impairments that determine one's ability to engage, disengage, and shift attention in coordination with fast-moving sensory cues. This supplementary analysis evaluated such a possibility, through a series of exploratory gaze data comparisons.

First, we examined whether there were any broad, autism-related differences in the frequency of gaze shifts during the study. The total number of saccades and fixations were assessed for each trial and averaged for both conditions, before being entered into separate mixed-model ANOVAs. Here, atypically-low frequencies might indicate impaired disengagement or shifting of visual attention. Conversely, any inaccuracies in continuous smooth pursuit or goal-directed saccades would likely demand a relatively *high* frequency of corrective gaze shifts[41]. Neither of these data patterns emerged, with ANOVAs showing null significant group (saccades: $F(1,70) = 2.10$, $p = .15$, $np^2 = .03$, $BF_{10} = .83$; fixations: $F(1,70) = .10$, $p = .75$, $np^2 = .001$, $BF_{10} = .44$), condition (saccades: $F(1,70) = 1.13$, $p = .29$, $np^2 = .02$, $BF_{10} = .37$; fixations: $F(1,70) = .80$, $p = .38$, $np^2 = .01$, $BF_{10} = .25$), and interaction effects (saccades: $F(1,70) = .11$, $p = .74$, $np^2 = .002$, $BF_{10} = .27$; fixations: $F(1,70) = .10$, $p = .75$, $np^2 = .001$, $BF_{10} = .28$). This suggests that autistic and neurotypical participants were shifting their gaze and fixating upon cues at a similar frequency in both conditions.

Next, we explored whether autism-related gaze differences in our task simply reflect impaired motion tracking abilities. If this was true, then we would expect particular difficulties to emerge on trials with the greatest ball velocities. As such, we extracted the average post-bounce distance (i.e., tracking error) between gaze and ball pitch vectors during all 'bouncy' ball trials. Here, any fundamental motion tracking impairments would result in generally high gaze-ball differences, regardless of whether the high-elasticity ball speeds are expected or uncertain. Therefore, we averaged bouncy-ball trial values from *both* conditions and compared them between groups using an independent t-test. Group averages were not statistically significant ($t(70) = .41$, $p = .68$, $BF_{10} = .27$), indicating that autistic and neurotypical participants had similar post-bounce tracking abilities with regards to the fast-moving ball cues.

Overall, this exploratory analysis finds little support for the notion that autism-related gaze differences in our study result from broad impairments in attentional and/or oculomotor control. Instead, they reinforce proposals that sensorimotor difficulties are likely related to context-sensitive mechanisms (e.g., trial-by-trial computations about likely ball bounciness probabilities and dynamic volatility estimations). Further empirical scrutiny is required, however, before any definitive conclusions can be made.

3. Exploratory Analyses of Gaze Fixation Variability

Autistic participants predictively positioned their gaze at a higher location than neurotypical individuals when the virtual balls were bouncing (Fig. 3). Such data patterns may be consistent with proposals that autistic people overestimate environmental volatility[31]: agents who perceive that the world is more changeable will increasingly update their long-term predictive models according to recent (high-elasticity) sensory information. Computationally, this would reflect an increase in learning rate[15], though such conclusions require further scrutiny (see main discussion).To initiate this enquiry, we conducted an exploratory analysis into the *variability* of participants' gaze fixation behaviours. Specifically, we looked at the standard deviation of bounce fixation locations (pitch angles) shown in each condition. If participant's visual sampling behaviours were being heavily driven by long-term prior expectations, then this trial-by-trial variability should be relatively low. On the other hand, larger standard deviations would indicate that gaze fixations are being strongly influenced by recent sensory data (i.e., changeable ball elasticity profiles from preceding trials).

ANOVA highlighted a significant effect of condition ($F(1,70) = 5.63$, $p = .02$, $np^2 = .07$, $BF_{10} = 3.03$) for these standard deviation values. Participants showed increased variability between stable and volatile conditions (Supplemetary Fig. S6), as indicative of an increased updating of prior models (i.e., a higher learning rate). While no significant interaction effects emerged ($F(1,70) < .001$, $p = .99$, $np^2 < .001$, $BF_{10} = .27$), the ASD group displayed generally higher trial-to-trial variability than their neurotypical counterparts ($F(1,70) = 38.47$, $p < .001$, $np^2 = .36$, $BF_{10} = 3.11*10^5$). This tendency to increasingly update bounce fixation locations on a trial-by-trial basis is in line with proposals that autistic people are over-reactive to environmental change and reinforces the potential role of aberrant volatility processing in ASD[31]. Research may wish to explore this topic further, by using sophisticated computational models of gaze fixation behaviours to estimate volatility-based learning rate parameters.

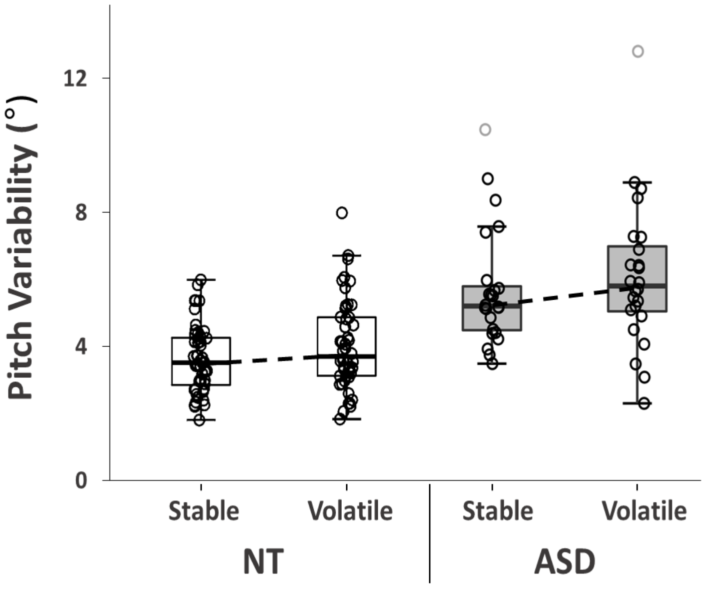

Supplementary Figure S6: Trial-by-trial standard deviation values corresponding to the spatial location (pitch angle, °) of bounce gaze fixations in each condition. NT: neurotypical; ASD: autism spectrum disorder. Two extreme values were identified and are represented as light grey circles (note: removal of these cases do not affected the overall pattern of results).
